# Elevated expression of mcl-1 inhibits apoptosis and predicts poor prognosis in patients with surgically resected non-small cell lung cancer

**DOI:** 10.1186/s13000-019-0884-3

**Published:** 2019-10-10

**Authors:** Qiuyuan Wen, Yuting Zhan, Hongmei Zheng, Hongjing Zang, Jiadi Luo, Yuting Zhang, Weiyuan Wang, Juan Feng, Junmi Lu, Lingjiao Chen, Songqing Fan

**Affiliations:** Department of Pathology, the Second Xiangya Hospital, Central South University, Changsha, 410011 Hunan China

**Keywords:** Non-small cell lung cancer (NSCLC), Mcl-1, Ki-67, C-PARP, Apoptosis

## Abstract

**Background:**

Mcl-1, an anti-apoptotic member of bcl-2 family, together with cleaved poly (ADC-ribose) polymerase (c-PARP) can serve as a marker of cell apoptosis. Previously we reported that treatment of Mnk inhibitor CGP57380 resulted in decreased Mcl-1 expression while increased c-PARP expression in non-small cell lung cancer (NSCLC) cells. In this study, we aimed to investigate association between Mcl-1 expression and clinicopathological features of NSCLC, and their correlation between Mcl-1 and both proliferation index (PI) and apoptotic index (AI) in NSCLC patients.

**Methods:**

Tissue microarrays (TMA) including 350 cases of surgically resected NSCLC were utilize and stained with Mcl-1, Ki-67 and c-PARP antibodies, PI and AI were then evaluated, respectively.

**Results:**

Higher Mcl-1 expression and PI were observed in NSCLC compared with non-cancerous lung tissues (non-CLT), while AI was significantly lower in lung adenocarcinoma (ADC) compared with non-CLT. Additionally, Mcl-1 expression in lung ADC was evidently higher than that of in lung squamous cell carcinoma (SCC). The elevated Mcl-1 expression was associated with PI, and inversely related to AI in NSCLC. NSCLC patients with elevated Mcl-1 expression and high PI, or with high Mcl-1 expression and low AI had remarkably shorter overall survival time than these patients with low Mcl-1 expression.

**Conclusions:**

Elevated expression of Mcl-1 might be inversely proportional to disease progression of NSCLC patients by promoting cell proliferation and inhibiting apoptosis, and Mcl-1 might serve as novel biomarker of poor prognosis for NSCLC patients.

**Supplementary information:**

**Supplementary information** accompanies this paper at (10.1186/s13000-019-0884-3).

## Background

Lung cancer represents one of the most common causes of cancer-related death worldwide. Non-small cell lung cancer (NSCLC), which includes squamous cell carcinoma (SCC) and adenocarcinoma (ADC), is the major histological subtypes and accounts for more than 80% of primary lung tumors [1]. Although the incidence of NSCLC is increasing due to improvement of early diagnosis and treatment modalities, its 5-year survival rate still remains very poor [2]. Therefore, there is an urgent need to identify new well-characterized biomarker(s) to improve clinical out-come of patients with NSCLC.

Myeloid cell leukemia 1 (Mcl-1), an anti-apoptotic member of the B-cell lymphoma 2 (bcl-2) family of apoptosis-regulating proteins, exemplifies a number of the mechanisms by which a protein’s contribution to cell fate may be modified [3]. Overexpression of Mcl-1 induces oncogenic transformation, and increased expression of Mcl-1 protein is found in the majority of human cancer including NSCLC. It is certified that Mcl-1 can promote cancer metastasis and resistance to conventional chemotherapy, and thus is correlated with poor prognosis [4, 5]. Mcl-1 inhibitor alone, or in combination with other inhibitors of key molecules presents a promising novel strategy to trigger cell death pathways in the treatment of cancer therapy [4, 6–9].

Members of poly ADP-ribose polymerase (PARP) enzymes family are ubiquitously expressed and involved in many key cellular processes such as genomic stability, adipocyte differentiation, DNA replication, DNA repair and cell death [10]. PARP could be cleaved by caspase-3, and cleaved PARP (c-PARP) is an early marker of chemotherapy-induced apoptosis in various cancer cells [11].

As a marker of proliferation in tumors, the Ki-67 proliferation index (PI) is routinely used in diagnosis of a wide range of cancers by measuring tumor proliferative activity. In addition, recent studies suggest that the PI might also have clinical impact in NSCLC [12, 13].

We previously reported that treatment of Mnk inhibitor CGP57380 with NSCLC cells results in different expression pattern of Mcl-1 and c-PARP, Mcl-1 expression is decreased whereas c-PARP is increased, the later plays an important role in inducing cell apoptosis through activating intrinsic mitochondrial pathway and represents cancer cell apoptotic index (AI). Furthermore, in vivo study indicates CGP57380 inhibits tumor growth as demonstrated by the sharp decline of PI in A549 cell xenograft mouse model [14]. These data suggested that Mcl-1 expression might tightly relate to PI and AI in NSCLC. However, there is no report about the correlations between the expression of Mcl-1 protein, PI, AI and the clinicopathologic/prognostic implication in large collection of NSCLC samples. In this retrospective study we investigated association between Mcl-1 expression and clinicopathological features of NSCLC, and their correlation between Mcl-1 and both PI and AI in NSCLC patients.

## Materials and methods

### Clinical data

350 cases of paraffin-embedded NSCLC as well as 53 cases of non-cancerous lung tissues (non-CLT) were obtained from Department of Pathology, the Second Xiangya Hospital of Central South University (Changsha, China). Clinicopathological data, including patient age, gender, clinical stages, lymph node status, histological type, pathological grade and so on, were shown in Additional file 1: Table S1. NSCLC patients had undergone clinical surgery at the Department of Thoracic Surgery at the Second Xiangya Hospital of Central South University. None of the patients received prior chemotherapy or radiotherapy before operation. The histological diagnosis and staging classification of patients were described in detail previously [14, 15]. In this study, we used the TMA technology designed and constructed high-throughput NSCLC TMAs according to rules previously described [15, 16].

### Immunohistochemistry and scores

The IHC staining for Mcl-1 protein, proliferation index (PI) with Ki-67 antibody and apoptotic index (AI) with c-PARP antibody in NSCLC TMAs was carried out using ready-to-use Envision TM+ Dual Link System-HRP methods (Dako, CA, USA). As described previously, the staining conditions for each antibody were adjusted according to our laboratory experience [15, 17, 18]. 1:300 dilution of primary antibody to Mcl-1 (Monoclonal Rabbit antibody, Catalog: #94296, Cell Signaling Technology), a 1:200 dilution of primary antibody to Ki-67 (Monoclonal Mouse antibody, Clone MIB-1, Dako) and 1:50 dilution of primary antibody to cleaved-PARP (Asp214) (Rabbit polyclonal antibody, #9541, Cell Signaling Technology) were applied to tissue sections to measure expression of the target proteins.

Staining were evaluated independently by two experienced pathologists. A semiquantitative evaluation of Mcl-1 expression was performed using a method described previously [15]. Staining intensity for Mcl-1 was scored as 0 (negative), 1 (weak), 2 (moderate), and 3 (strong). The percentage of positive cells was divided into five grades (percentage scores): 0 (0%), 1 (1–25%), 2 (26–50%), 3 (51–75%), and 4 (76–100%). Staining positivity was determined by the formula: overall scores = percentage score × intensity score. The result of the staining scores was used as the final staining score for Mcl-1 (0–12). An optimal cut-off level for Mcl-1 was chosen on the basis of a measure of heterogeneity using the log-rank test with respect to overall survival (OS). The final staining score of 5–7 was considered to be high expression of Mcl-1 while scores less than 5 were considered low expression of Mcl-1. As for Ki-67 staining, it was regarded high PI when the percentage of cells were more than 15% positive staining, and if not, it was regarded as low PI. C-PARP expression was estimated microscopically by counting c-PARP positive cells at original magnification × 400 using the semi-quantitative method described in the literature with minor modification as follows: negative (−, no positive cells), weak (+, 1–5 positive cells), moderate (++, 5–15 positive cells) and strong (+++, > 15 positive cells) in views obtained with the area of 10 high power fields [19]. More than 5 positive cells were considered to be high AI while less than 5 was considered low AI. Agreement between the two evaluators was 98%, and all scoring discrepancies were resolved through discussion between the two evaluators.

### Statistical analysis

All statistical analysis was performed by SPSS 23.0. The chi-square test and the Spearman’s rank correlation coefficient were used to evaluate the relationship between the Mcl-1 expression, PI and AI in NSCLC. Kaplan-Meier analysis was performed for overall survival curves and statistical significance was assessed using the log-rank test. Overall survival was defined as the time from the treatment initiation (diagnosis) to the date of death. Cox proportional hazard regression model was used to estimate the independent prognostic factor Mcl-1, PI and AI. Two-sided statistical analysis was used and the data were considered to be statistically significant when *P* < 0.05.

## Results

### Association between mcl-1 expression, PI and AI and clinicopathological features of NSCLC

**Fig. 1:**
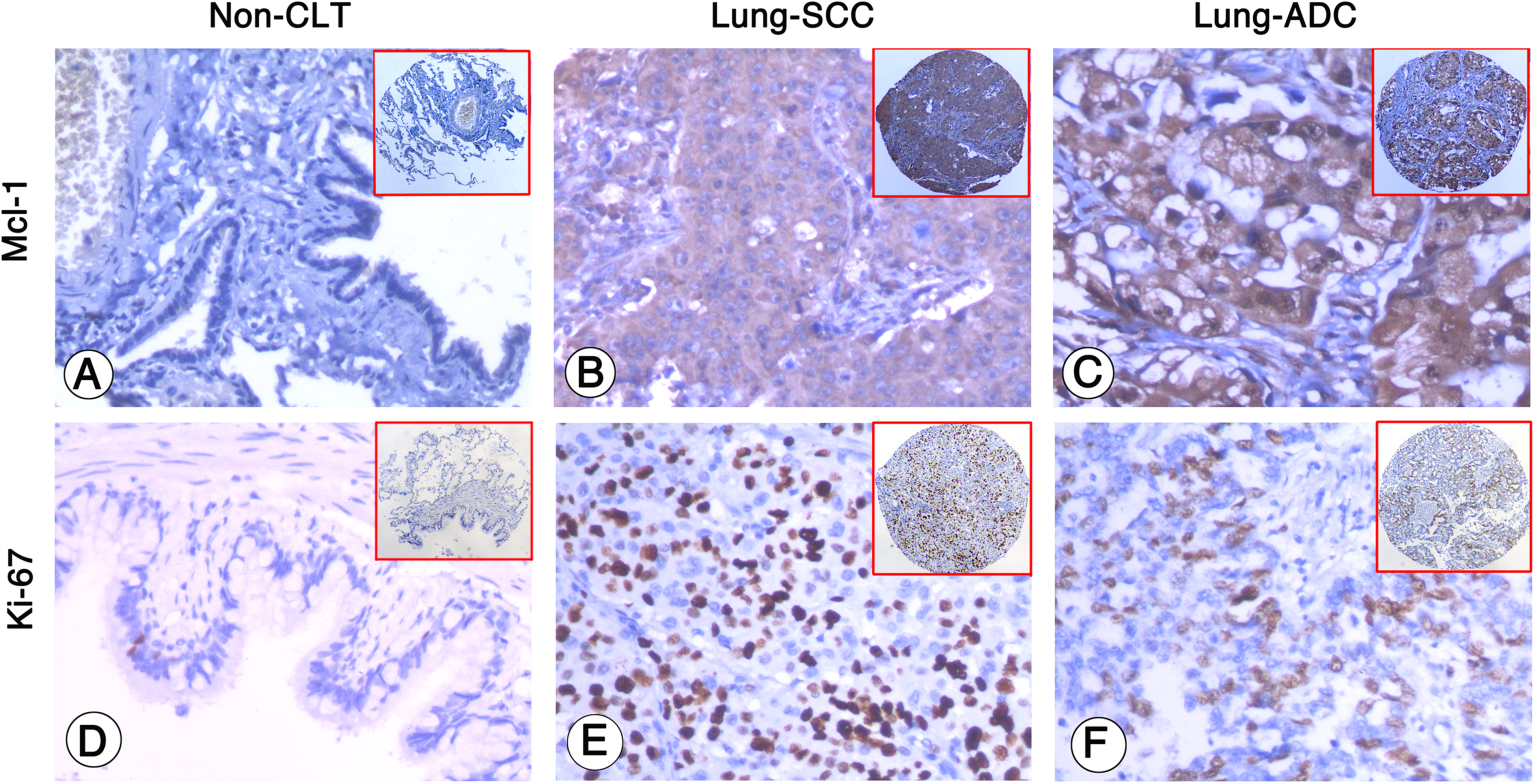
Mcl-1 expression and Ki-67 PI in non-cancerous lung tissues (non-CLT), lung SCC cells and lung ADC cells were detected by IHC using specific antibody as described in the section of materials and methods. Negative staining of Mcl-1 and low PI were showed in non-CLT (**a** and **d**, 200×, IHC, DAB staining). Strong positive staining of Mcl-1 was found in cell cytoplasm of lung SCC and lung ADC cells (**b** and **c**, 200×, IHC, DAB staining), and high PI was found in the nucleus of lung SCC and lung ADC cells (**e** and **f**, 200×, IHC, DAB staining)

**Fig. 2:**
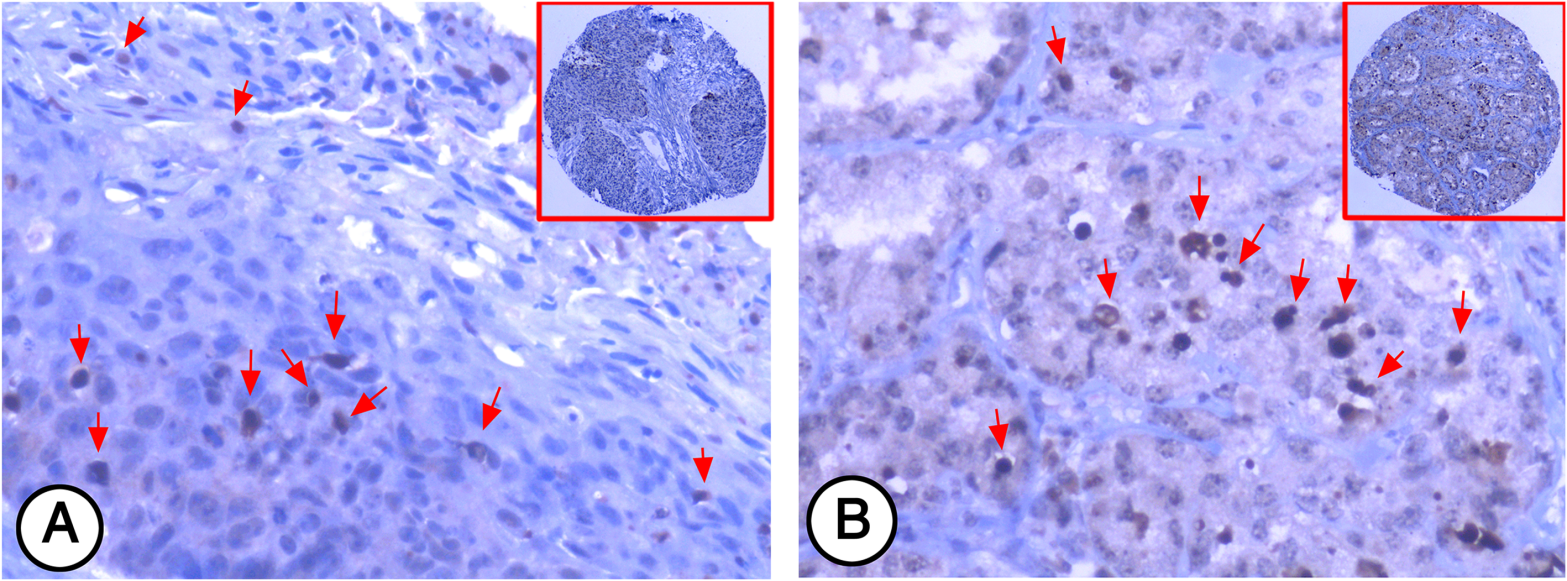
C-PARP AI in NSCLC was detected by IHC. Positive staining of c-PARP (arrows) was found in the nucleus of lung SCC and ADC cells (**a** and **b**, 200×, IHC, DAB staining)

Strong Mcl-1 expression (Fig. 1b and c) was found in cytoplasm of lung SCC and ADC cells while no Mcl-1 expression was detected in non-CLT (Fig. 1a). PI was identified in the nucleus of Ki-67 positive lung cancer cells. High PI (Fig. 1e and f) was found in the lung SCC and ADC tissues, in contrast, low PI was found in non-CLT (Fig. 1d). Similarly, AI was found in the nucleus of c-PARP positive lung cancer cells. As shown in Fig. 2a and b, lung SCC and ADC cells displayed positive staining of c-PARP in the nucleus represent the AI was identified.

**Fig. 3:**
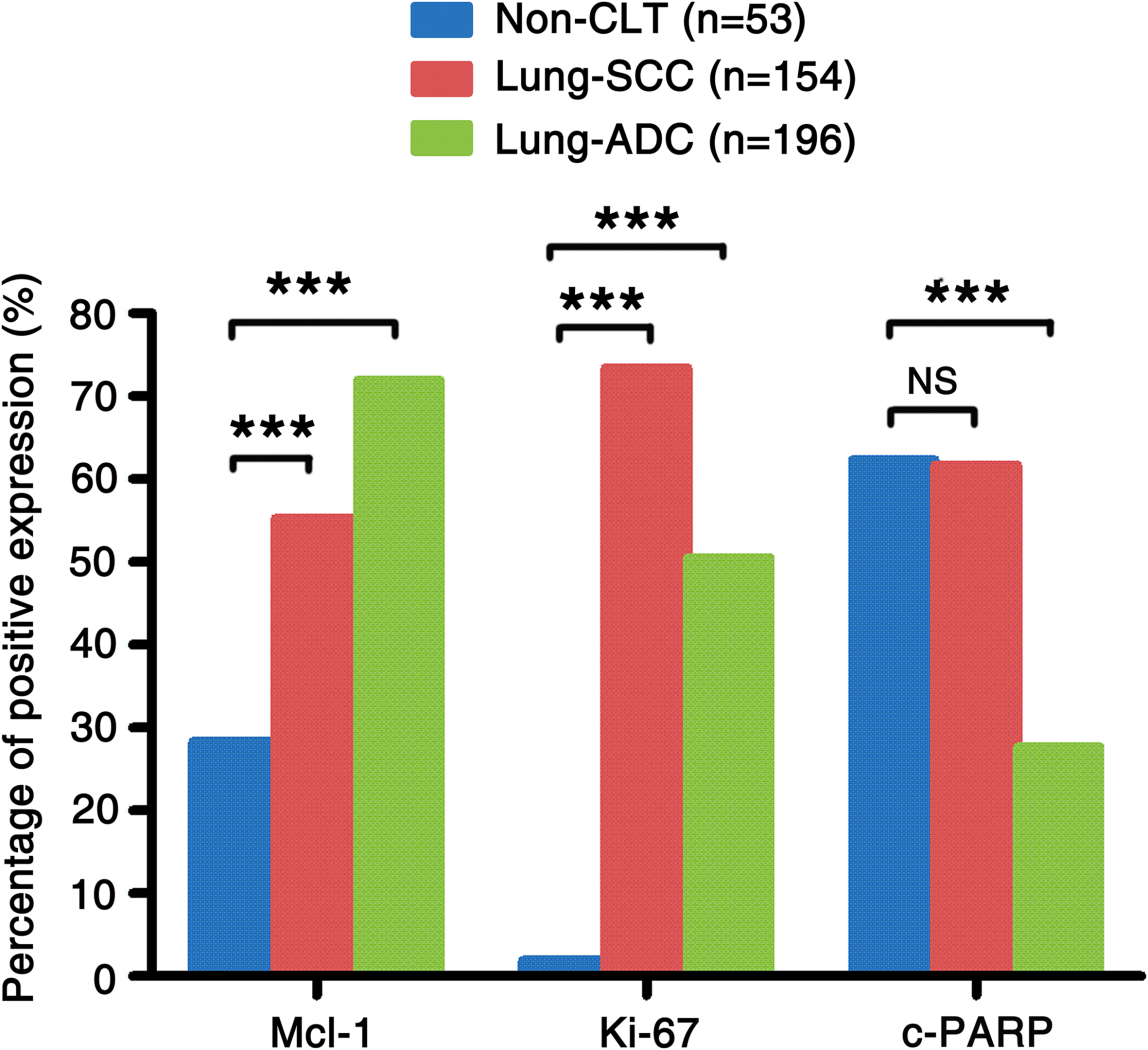
Mcl-1 expression, Ki-67 PI and c-PARP AI in lung SCC and lung ADC compared to non-CLT. Results showed that there were significant differences between the groups which were statistically by chi-square test (***P < 0.001). No apparent difference in AI was found between lung SCC and non-CLT (*P* > 0.05)

Furthermore, Mcl-1 expression and PI were significantly higher in lung SCC and ADC compared with non-CLT (*P* < 0.001). In contrast, AI was obviously lower in lung ADC compared with non-CLT (*P* < 0.001). There was no statistical difference in AI between lung SCC and non-CLT (*P* > 0.05) (Fig. 3).

We further examined correlation between high Mcl-1 expression and the histological type of NSCLC, data were shown in Table 1. Our analysis indicated that there is a strong positive correlation between high Mcl-1 expression and the histological type of NSCLC. Patients with ADC had significantly higher Mcl-1 expression than patients with SCC (*P* = 0.002). However, inverse trend (Table 1) was observed in case of PI and AI (*P* < 0.001, *P* < 0.001, respectively). Of note, higher PI and AI was found in male than in female (*P* = 0.007, *P* = 0.001, respectively). Furthermore, high Mcl-1 expression (*P* < 0.001) or low AI (*P* < 0.001) had an evidently inverse correlation with survival status of NSCLC patients. A conjoint analysis also indicated that patients with high Mcl-1 expression and PI, or with high Mcl-1 expression and low AI suffered a lower overall survival rates than that with other phenotypes of Mcl-1, PI and AI (*P* = 0.002, *P* < 0.001, respectively). No differences were observed between Mcl-1 expression/PI/AI and other clinicopathological features such as age, LNM status, clinical stages and pathological differentiation of NSCLC patients.

**Table 1:** Analysis of the association between expression of Mcl-1 and Ki-67 PI and c-PARP AI and clinicopathological features of NSCLC (*n* = 350)

Clinicopathological features (n)	Mcl-1			PI			AI			Mcl-1/PI ^#^			Mcl-1/AI^#^		
	High (%)	Low (%)	*P*-value	High (%)	Low (%)	P-value	High (%)	Low (%)	P-value	P^+^ (%)	N^−^ (%)	P-value	P^+^ (%)	N^−^ (%)	*P*-value
Age (years)															
≤ 50 (*n* = 95)	62 (65.3)	33 (34.7)		52 (54.7)	43 (45.3)		37 (38.9)	58 (61.1)		34 (35.8)	61 (64.2)		42 (44.2)	53 (55.8)	
>50 (*n* = 255)	164 (64.3)	91 (35.7)	.901	160 (62.7)	95 (37.3)	0.178	112 (43.9)	143 (56.1)	0.466	115 (45.1)	140 (54.9)	0.114	103 (40.4)	152 (59.6)	0.543
Gender															
Male (*n* = 266)	171 (64.3)	95 (35.7)		172 (64.7)	94 (35.3)		127 (47.7)	139 (52.3)		121 (45.5)	145 (54.5)		102 (38.3)	164 (61.7)	
Female (*n* = 84)	55 (65.5)	29 (34.5)	0.896	40 (47.6)	44 (52.4)	0.007*	22 (26.2)	62 (73.8)	0.001*	28 (33.3)	56 (66.7)	0.058	43 (51.2)	41 (48.8)	0.042*
Clinical stages															
Stage _I-II_ (*n* = 151)	91 (60.3)	60 (39.7)		95 (62.1)	56 (37.1)		69 (45.7)	82 (54.3)		61 (40.4)	90 (59.6)		56 (37.1)	95 (62.9)	
Stage _III_ (*n* = 199)	135 (67.8)	64 (32.2)	0.145	117 (58.8)	82 (41.2)	0.442	80 (40.2)	119 (59.8)	0.327	88 (44.2)	111 (55.8)	0.513	89 (44.7)	110 (55.3)	0.156
LN status															
LNM (*n* = 210)	137 (65.2)	73 (34.8)		127 (60.5)	83 (39.5)		86 (41.0)	124 (59.0)		90 (42.9)	120 (57.1)		97 (43.3)	119 (56.7)	
No LNM (*n* = 140)	89 (63.6)	51 (36.4)	0.820	85 (60.7)	55 (39.3)	1.000	63 (45.0)	77 (55.0)	0.508	59 (42.1)	81 (57.9)	0.913	54 (38.6)	86 (61.4)	0.438
Histological type															
SCC (*N* = 154)	85 (55.2)	69 (44.8)		113 (73.4)	41 (26.6)		95 (66.7)	59 (83.3)		71 (46.1)	83 (53.9)		37 (24.0)	117 (76.0)	
ADC (*N* = 196)	142 (71.9)	55 (28.1)	0.002*	99 (50.5)	97 (49.5)	0.000*	54 (27.6)	142 (72.4)	0.000*	78 (39.8)	118 (60.2)	0.276	108 (55.1)	88 (44.9)	0.000*
Pathological grade															
Well (*n* = 6)	6 (100.0)	0 (0.0)		2 (33.3)	4 (66.7)		1 (16.7)	5 (83.3)		2 (33.3)	4 (66.7)		5 (83.3)	1 (16.7)	
Moderate (*n* = 146)	94 (66.4)	52 (35.6)		88 (60.3)	58 (39.7)		61 (41.8)	85 (58.2)		58 (39.7)	88 (60.3)		65 (44.5)	81 (55.5)	
Poor (*n* = 198)	126 (63.6)	72 (36.4)	0.185	122 (61.6)	76 (38.4)	0.375	87 (43.9)	111 (56.1)	0.399	89 (44.9)	109 (55.1)	0.562	75 (37.9)	123 (62.1)	0.051
Survival status															
Alive (*n* = 187)	105 (56.1)	82 (43.9)		113 (60.4)	74 (39.6)		97 (51.9)	90 (48.1)		65 (34.8)	122 (65.2)		60 (32.1)	127 (67.9)	
Dead (*n* = 163)	121 (74.2)	42 (25.8)	0.000*	99 (60.7)	64 (39.3)	1.000	52 (31.9)	111 (68.1)	0.000*	84 (51.5)	79 (48.5)	0.002*	85 (52.1)	78 (47.9)	0.000*

*Chi-square test, statistically significant difference (*P* < 0.05). Abbreviations: *LNM* lymph node metastasis, *SCC* squamous cell carcinoma, Mcl-1/PI^#^P^+^, common high expression of Mcl-1 and Ki-67 PI, P^−^, either low expression of the two proteins, *Mcl-1/AI*^*#*^*P*^*+*^ high expression of Mcl-1 and low *c-PARP AI P*^*−*^, other combination of expression of this two proteins

### The pairwise association between expression of mcl-1, PI and AI in NSCLC

Data in Additional file 2: Table S2. suggested that elevated Mcl-1 expression was significantly associated with high PI, while it was evidently negative related to high AI in NSCLC (r = 0.148, *P* = 0.006, and r = − 0.184, *P* = 0.001, respectively), indicating aberrant high Mcl-1 expression might play an important role in inhibiting apoptosis and promoting cell survival in NSCLC.

### Impact of mcl-1 expression, PI and AI on the prognosis of patients with NSCLC

**Fig. 4:**
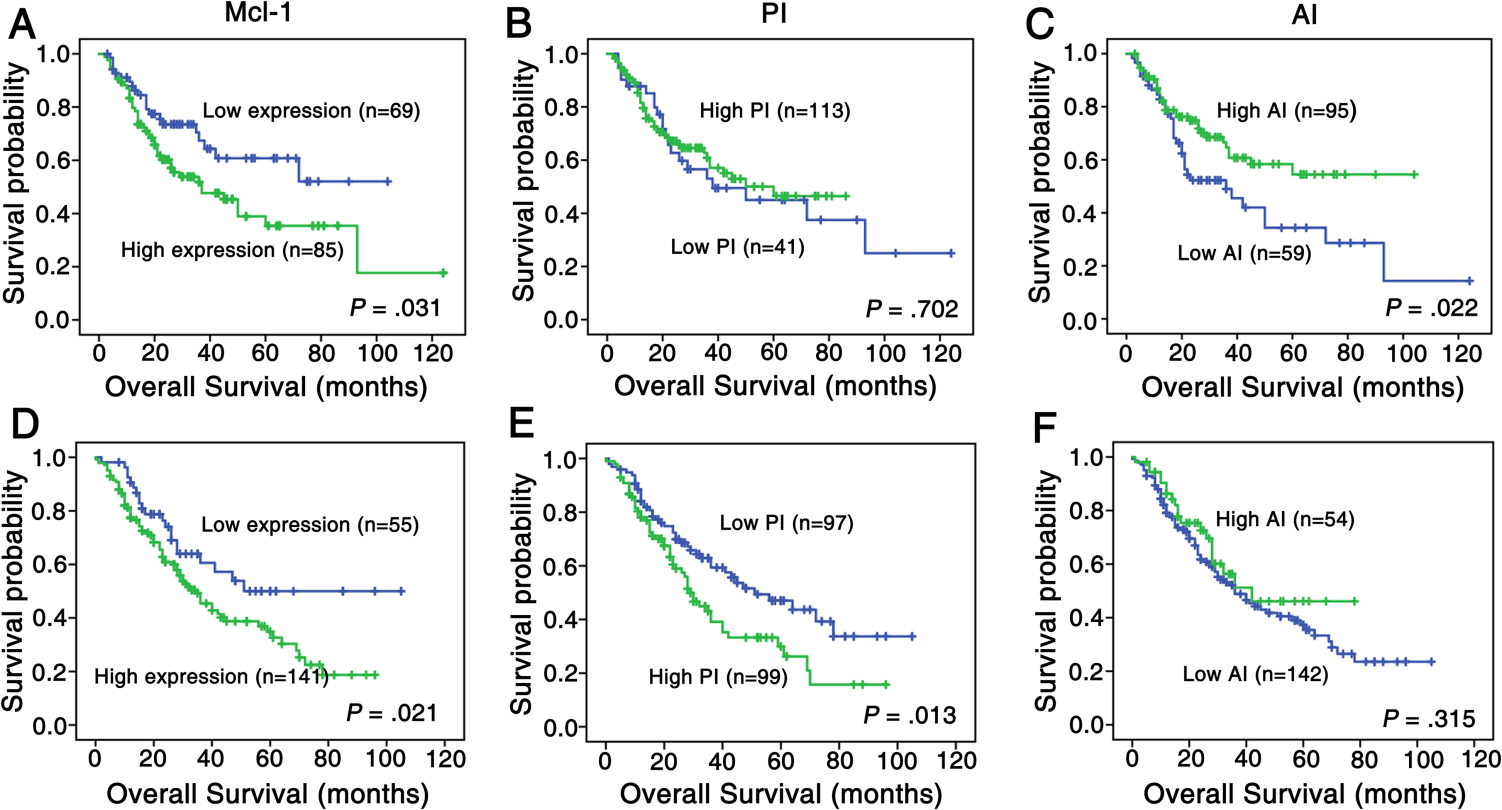
Kaplan-Meier cures for overall survival of lung SCC and lung ADC patients with Mcl-1 expression, PI and AI. Kaplan-Meier analysis was used to plot the overall survival of 154 cases of lung SCC (**a**-**c**) and 196 cases of lung ADC (**d**-**f**) patients with differential Mcl-1 expression, PI and AI, which statistical significance was assessed by log-rank test. (**a**) Lung SCC patients with high Mcl-1 expression showed worse overall survival rates compared to patients with low Mcl-1 expression (*P* = 0.031, two sided). (**b**) PI had no significantly correlation with overall survival rates of lung SCC patients (*P* > 0.05, two sided). (**c**) Lung SCC patients with high AI showed better overall survival rates compared to those with low AI (*P* = 0.022, two sided). (**d**) Lung ADC patients with high Mcl-1 expression had worse overall survival rates than that with low Mcl-1 expression (*P* = 0.021, two sided). (**e**) Lung ADC patients with high PI showed worse overall survival rates than those with low PI (*P* = 0.013, two sided). (**f**) The AI had no significantly correlation with overall survival rates of lung ADC patients (*P* > 0.05, two sided)

**Fig. 5:**
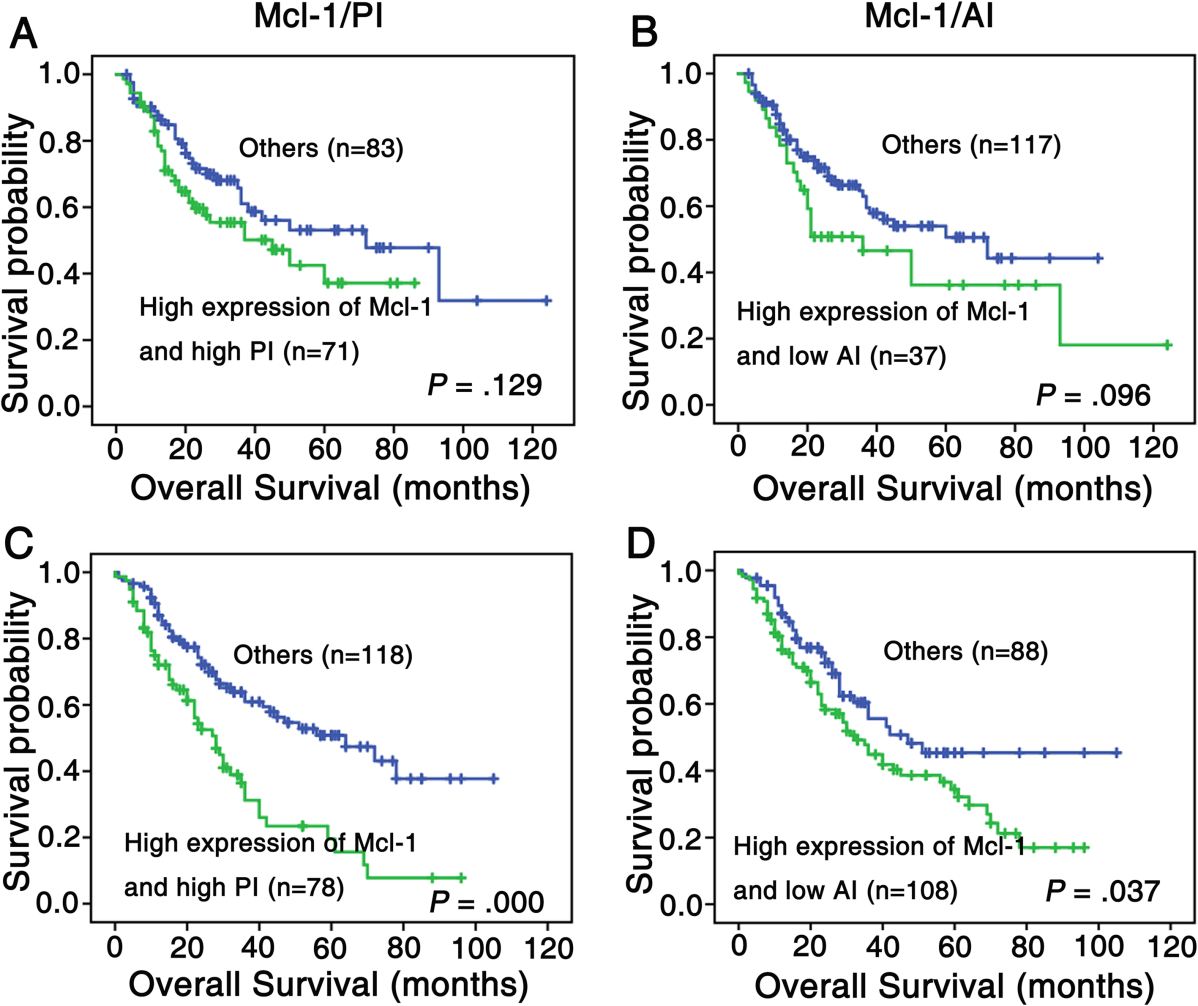
Kaplan-Meier analysis was used to plot the overall survival of 154 cases of lung SCC (**a** and **b**) and 196 cases of lung ADC (**c** and **d**) patients with differential combined Mcl-1 expression, PI and AI, which statistical significance was assessed by log-rank test. (**a**) Kaplan-Meier curves showed that there was no statistical significance between lung SCC patients with combined high Mcl-1 expression and PI and that with low expression with either of two proteins mentioned above (*P* > 0.05, two sided). (**b**) There was no apparent difference between lung SCC patients with combined high Mcl-1 expression and low AI and that with other expression patterns of these two proteins (*P* = 0.096, two sided). (**c**) Lung ADC patients with high expression of both Mcl-1 and PI had worse overall survival rates compared with either of low expression of two proteins above (*P* < 0.0001, two sided). (**d**) Lung ADC patients with high Mcl-1 expression and low AI owned poor prognosis compared with that with other expression patterns of these tow proteins (*P* = 0.037, two sided)

We then run univariate survival analysis in these lung cancer patients. Kaplan-Meier survival curve analysis with log-rank significance test was performed. Figure 4 illustrated the Kaplan-Meier survival plots for lung SCC patients and lung ADC patients with different Mcl-1 expression (Fig. 4a and d), PI (Fig. 4b and e), AI (Fig. 4c and f) and combination of Mcl-1, PI and AI were presented in Fig. 5. The overall survival rates were significantly higher in lung SCC and ADC patients with low Mcl-1 expression than these with high Mcl-1 expression (*P* = 0.031, Fig. 4a; *P* = 0.021, Fig. 4d respectively). Moreover, patients had worse prognosis with high PI or low AI (*P* = 0.013 for ADC, Fig. 4e; *P* = 0.022 for SCC, Fig. 4c, respectively); also, lung ADC patients with high Mcl-1 expression and PI, or with high Mcl-1 expression and low AI had significantly lower overall survival rates than patients with any other patterns of Mcl-1/PI/AI (*P* = 0.000, Fig. 5c; *P* = 0.037, Fig. 5d). There were no significant associations between PI alone, high Mcl-1 expression as well as PI and overall survival rates were noticed in lung SCC patients.

We further investigated whether the expression of Mcl-1, PI and AI can be used as an independent prognostic factor for NSCLC. As shown in Table 2, high Mcl-1 expression and high PI may serve as an independent poor prognostic factors for lung ADC (*P* = 0.035, *P* = 0.029, respectively), as well as clinical stages (*P* = 0.023). For lung SCC, pathological grades and LNM status are identified as independent poor prognostic factors (*P* = 0.010, *P* = 0.003, respectively). No clinical effect was detected with age, gender, treatment strategy in lung SCC and ADC (*P* > 0.05 for all).

**Table 2:** Summary of multivariate of Cox proportional regression for overall survival in 350 cases of NSCLC

Histological type	Lung SCC				Lung ADC			
Parameter	Sig.	Exp (B)	95.0% CI for Exp (B)		Sig.	Exp (B)	95.0% CI for Exp (B)	
			Lower	Upper			Lower	Upper
Age	0.705	1.121	0.621	2.024	0.672	0.909	0.584	1.414
Gender	0.059	0.141	0.019	1.079	0.529	0.873	0.572	1.333
Pathological grade	0.010*	2.149	1.208	3.835	0.252	1.247	0.854	1.821
Clinical stages	0.115	1.699	0.879	3.286	0.023*	1.740	1.080	2.804
LNM status	0.003*	2.523	1.357	4.688	0.084	1.546	0.943	2.534
Treatment strategy	0.724	0.907	0.529	1.558	0.948	1.013	0.685	1.499
Mcl-1 expression	0.655	1.139	0.643	2.020	0.035*	1.724	1.038	2.864
AI	0.105	0.637	0.369	1.099	0.235	0.733	0.439	1.225
PI	0.263	1.434	0.763	2.696	0.029*	1.596	1.049	2.429

Abbreviations: *LNM* lymph node metastasis, *SCC* squamous cell carcinoma, *ADC* adenocarcinoma, *CI* confidence interval. Note: multivariate analysis of Cox regression, *P < 0.05

## Discussion

Members of bcl-2 family represented a new class of oncogene by maintaining viability through inhibition of apoptosis, whose dysregulation was involved in virtually all malignancies, and a number of other pathologies [3]. Mcl-1 overexpression has been found in several hematological cancers and solid tumors, including chronic myeloid leukemia, gastric cancer and lung cancer [20–23]. In this study, we found that Mcl-1 expression and PI were remarkably increased in lung SCC and ADC compared with non-CLT, which is in accordance with several studies reported by other investigators [20]. In addition, our data showed that increased Mcl-1 expression was significantly associated with high PI and negatively related to high AI in NSCLC. The results suggest that elevated expression of Mcl-1 might be involved in inhibiting apoptosis and promoting cell survival in NSCLC.

We showed that both lung SCC and ADC patients had worse overall survival rates with increased Mcl-1 expression compared with low Mcl-1 expression, lung ADC patients with high Mcl-1 expression and low AI have a far worse prognosis compared to patients with other immunophenotype of Mcl-1 and AI. Furthermore, multivariate analysis proved that increased Mcl-1 expression was an independent factor for poor prognosis in lung ADC patents. Of note, our results suggest that high Mcl-1 expression might participate in inhibiting cell apoptosis and associate with the poor prognosis of lung ADC patients. Therefore, increased Mcl-1 expression might be used as a novel biomarker to predict poor prognosis for lung ADC patients. Our results provide evidence that inhibiting Mcl-1 would be a promising novel strategy to trigger cell death pathways in the treatment of NSCLC therapy.

The correlation between PI and prognosis of neoplasm is reported in many tumors including lung cancer [24]. Several studies revealed that PI often fails to be an independent prognostic factor in multivariate analyses, or as a negative association to prognosis in lung cancer [25–27]. In our study, high PI had significant correlation with overall survival rates for lung ADC patients. Furthermore, lung ADC patients with high Mcl-1 expression and high PI had lower overall rates than those with low Mcl-1 expression or PI. Our studies demonstrated surgically resented NSCLC patients with high Mcl-1 expression and high PI had poor overall survival, which suggest that Mcl-1 and high PI might have a positive synergistic effect on patients’ outcome.

Dysregulated apoptosis plays a central role in cancer development and limits the efficacy of conventional cytotoxic therapies [28–30]. Evidence is accumulating that Mcl-1 could decide cell fate by changes in transcription, localization, stability and its ability to form dimers with bcl-2 homologues and other proteins. Moreover, it is demonstrated that patients with solid tumors and leukemia benefit from decreased Mcl-1 expression or reducing its stability [31]. Thus Mcl-1 is an attractive and potential therapeutic target in a number of malignancies, and also plays an important role in the resistance to anticancer therapies [4, 21, 32, 33]. So far, many Mcl-1 protein inhibitors such as Mcl-1 antisense oligonucleotides, staple-peptides and small-molecule inhibitors have been reported, but there still be a long way to go before they are put into clinical practice [34]. The underlying mechanisms and effects of the up-expression of Mcl-1 in NSCLC are not clearly elucidated yet. A recent study shows that Mcl-1 can be cleaved by caspase-3 in NSCLC cells that are undergoing chemotherapeutic agent-triggered apoptosis. The stability of cleaved Mcl-1 supports the correlation between Mcl-1 expression and the relative resistance of NSCLC patients to chemotherapy [35]. Our previous study showed that treatment of Mnk inhibitor CGP57380 resulted in decreased Mcl-1 expression while increased c-PARP expression in NSCLC cells. Taking together, our data highlighted the role of Mcl-1 might play in apoptosis of NSCLC cells [14]. Obviously, further studies and more researches are in need to elucidate the precise mechanism of the role of Mcl-1 protein in NSCLC.

## Conclusions

The expression of Mcl-1 and PI significantly increased in lung SCC and lung ADC tissues, and AI obviously decreased in lung ADC tissues. High Mcl-1 expression might promote cell proliferation and inhibit apoptosis, which correlated with poor prognosis for NSCLC patients, and Mcl-1 might serve as novel biomarker of poor prognosis in surgically resected NSCLC patients.

## Supplementary information

**Additional file 1: Table S1.** Clinicopathological features of patients with NSCLC and non-cancerous control lung tissues. (DOCX 29 kb)

**Additional file 2: Table S2.** The pairwise association between expression of Mcl-1, PI and AI in 350 cases of NSCLC. (DOCX 27 kb)

## Data Availability

All data for this study are presented in the manuscript.

## Abbreviations

ADC: Adenocarcinoma
AI: Apoptotic Index
IHC: Immunhistochemistry
Non-CLT: Non-Cancerous Lung Tissues
NSCLC: Non-small Cell Lung Cancer
PARP: Poly ADP-ribose Polymerase
PI: Proliferation index
SCC: Squamous Cell Carcinoma
TMA: Tissue microarrays
WHO: World Health Organization
