# Supplementary material for: Elevated expression of mcl-1 inhibits apoptosis and predicts poor prognosis in patients with surgically resected non-small cell lung cancer
Source: Diagn Pathol. 2019 Oct 10;14:108. doi: 10.1186/s13000-019-0884-3 (PMC6788105; doi:10.1186/s13000-019-0884-3)
Supplement: Supplementary file 1 — Additional file 1: Table S1. Clinicopathological features of patients with NSCLC and non-cancerous control lung tissues. (DOCX 29 kb) [file 13000_2019_884_MOESM1_ESM.docx]

**Additional file 1: Table S1. Clinicopathological features of patients with NSCLC and non-cancerous control lung tissues**

| **Patients characteristics** | No. of patients (%) |
| --- | --- |
| **NSCLC** |  |
| **Age(years)** |  |
| ≤50 | 95(27.1) |
| ＞50 | 255(72.9) |
| **Gender** |  |
| Male | 266(76.0) |
| Female | 84(24.0) |
| **Clinical stages** |  |
| Stage _Ⅰ_ | 76(21.7) |
| Stage _Ⅱ_ | 75(21.4) |
| Stage _Ⅲ_ | 199(56.9) |
| **Lymph node status** |  |
| N0 | 140(40.0) |
| N1/N2/N3 | 210(60.0) |
| **Histological type** |  |
| SCC | 154(44.0) |
| ADC | 196(56.0) |
| **Pathological grade** |  |
| Well | 6(1.7) |
| Moderate | 146(41.7) |
| Poor | 198 (56.6) |
| **Non-cancerous control lung tissues** |  |
| **Age(years)** |  |
| ≤50 | 22(41.5) |
| ＞50 | 31(58.5) |
| **Gender** |  |
| Male | 27(50.9) |
| Female | 26(49.1) |
