# Supplementary material for: Elevated expression of mcl-1 inhibits apoptosis and predicts poor prognosis in patients with surgically resected non-small cell lung cancer
Source: Diagn Pathol. 2019 Oct 10;14:108. doi: 10.1186/s13000-019-0884-3 (PMC6788105; doi:10.1186/s13000-019-0884-3)
Supplement: Supplementary file 2 — Additional file 2: Table S2. The pairwise association between expression of Mcl-1, PI and AI in 350 cases of NSCLC. (DOCX 27 kb) [file 13000_2019_884_MOESM2_ESM.docx]

**Additional file 2: Table S2. The pairwise association between expression of Mcl-1, PI and AI in 350 cases of NSCLC**

|  |  | **PI** |  |  | **AI** |  |
| --- | --- | --- | --- | --- | --- | --- |
|  | High (%) | Low (%) | *P*-value | High (%) | Low (%) | *P*-value |
| **Mcl-1** |  |  |  |  |  |  |
| High (%) | 149(65.9) | 77(34.1) | .006 | 81(35.8) | 145(64.2) | .001 |
| Low (%) | 63(50.8) | 61(49.2) | (r = 0.148) | 68(54.9) | 56(45.1) | (r = -0.184) |

*Spearman’s rank correlation test, statistically significant difference (*P* < 0.05)
